# What the Local Centres Are Doing

**Published:** 1921-03-19

**Authors:** 


### COLLEGE OF NURSING

(Limited by Guarantee.)

### What the Local Centres are Doing.

### LONDON CENTRE.

(Secretary, Miss Bompas, 7 Henrietta Street, W. 1.)

Notices.

Members of the London Centre of the College of Nursing are reminded that their subscription of 5s. for the year 1921-1922 is due on April 1, and they are asked to forward it at their earliest convenience to the Secretary of the London Centre as above.

The London Centre Club Rooms will be closed from Thursday night, March 24, to Tuesday morning, March 29.

Nominations: A Warning.

The Returning Officer reports that many nomination papers are being returned unsigned by the nominated candidate, and warns members that papers so returned are invalid, and, therefore, useless.

Women and the Vote.

At the lecture to members of the London Centre on Thursday, March 10, by Miss Eleanor Rathbone, C.C., J.P., on "The Vote and What it has Done for Women," Miss Rathbone so impressed her audience with the importance of equal citizenship for men and women that the following resolution was passed, and has been forwarded to the Prime Minister: "That this meeting of the London Centre of the College of Nursing urges the Government to redeem its election pledges by introducing a measure extending the Parliamentary Franchise to women upon the same terms as men."

Election Organisation.

A meeting of members was held at the College of Ambulance, 56 Queen Anne Street, W., on Tuesday, March 15, to consider the question of the policy to be adopted in connection with nominations for the Council election. Miss Montgomery was in the chair. Mrs. Oliver Strachey gave an excellent address on "Election Organisation," which cleared the ground, and the meeting then proceeded to apply the principles she enunciated. The result will be found in the article entitled "The College Nominations" on page 567.

### BRIGHTON AND HOVE CENTRE.

(Hon. Secretary, Miss A. M. Latham, Royal Sussex County Hospital.)

Wednesday, March 30, 7.30 p.m.—At the College of Nursing Club, No. 45 Marine Parade, members' meeting to discuss and to decide whether (*a*) professional women other than nurses, (*b*) nurses in training, be admitted as members and as residential visitors to the Club; and if professional women are admitted, which professions shall be included? The meeting will also elect new members in place of the retiring members on the Executive Committee, and a President, Hon. Secretary, and Hon. Treasurer.

### BIRMINGHAM THREE COUNTIES CENTRE.

(Hon. Secretary, Mrs. Glegg, 84 Hagley Road, Edgbaston.)

Tuesday, March 22, 5.30 p.m.—In the Lecture Theatre, General Hospital, Birmingham (by kind permission of the Governors), Dr. Arthur Loxton will lecture on "Some Diseases of the Skin." Admission, members free; non-members entrance, 1s.

### THE YORKSHIRE CENTRE.

(Hon. Secretary, Miss M. V. Lindall, Hospital for Women and Children, Leeds.)

Thursday, March 31, 7 to 10.30 p.m.—Members' meeting at the Leeds General Infirmary, and whist-drive, by kind invitation of Miss Innes, R.R.C. R.S.V.P. to Miss Innes on or before Saturday, March 26, at latest. Admission at the main entrance of the Infirmary.

Notice.

The Hon. Secretary reminds the members that the annual subscription for the Centre has been raised to 5s. this year, and is due on April 1.

